# Women and HIV: the urgent need for more research and policy attention in the Middle East and North Africa region

**DOI:** 10.7448/IAS.18.1.20084

**Published:** 2015-03-08

**Authors:** Jocelyn DeJong, Francesca Battistin

**Affiliations:** Faculty of Health Sciences, American University of Beirut, Beirut, Lebanon

HIV surveillance systems and research in the Middle East and North Africa (MENA) region have improved markedly over the last decade [1] but nevertheless are challenged by inadequate detection of new infections and under-reporting of known infections [2,3]. There are indications, however, that there may be a specific risk of under-detection of HIV among women. Given that sexual behaviour is deeply rooted in gender norms, it is critical to understand how these shape risk behaviour and vulnerability to HIV infection in specific contexts. Until recently, HIV surveillance efforts internationally have not integrated gender considerations into their methods, sampling and tools, and this certainly applies in the MENA region [4]. Identifying the barriers to HIV case detection among women in the region is critical to improving women's health, providing them with appropriate access to prevention and treatment services, as well as preventing onward transmission of HIV.

In the most comprehensive assessment of the epidemiology of HIV infection in the MENA region, Abu-Raddad and colleagues conclude that until recently men account for the vast majority of infections, and that the overwhelming risk factor for women to acquire HIV in the region is marriage [5]. Bozicevic, Riedner and Calleja compare case-reporting data submitted by 18 Arab countries (recalculated in Table 1 below) for the year 2010 [2]. The average male-to-female ratio is 3, four countries have a male-to-female ratio of HIV/AIDS of 1: 2, nine countries have a ratio of 2 and four countries have a ratio of over 5. As they argue, this suggests either that substantial HIV transmission might have occurred via male-to-male sex, or that female HIV cases are underreported. Yet in 8 out of the 16 countries, the most commonly reported mode of transmission in men is heterosexual and only in Lebanon is transmission among men the most frequently reported transmission mode [2]. In settings with heterosexual transmission of HIV, one would expect a much lower male/female ratio. In sub-Saharan Africa, women are disproportionately more infected than males [6]. It should be noted, moreover, that the MENA region is one in which male circumcision is very high, across religious groups, which is a differential protective factor favouring men [5]. There is clearly a need for more research to investigate the reasons for such gender ratios.

**Table 1:** Gender ratios (from highest to lowest) for 2010 of reported HIV and AIDS cases, selected Arab countries

Country	Male HIV cases	Male AIDS cases	Total male HIV/AIDS	Female HIV cases	Female AIDS cases	Total female HIV/AIDS	Male/Female ratio HIV/AIDS cases^a^
Bahrain	14	NA	14	2	NA	2	7.0
Lebanon	18	22	40	3	4	7	5.7
Jordan	9	b	16	3	0	3	5.3
United Arab Emirates	21	NA	21	4	NA	4	5.3
Saudi Arabia	309	44	353	77	9	86	4.1
Egypt	326	NA	326	83	NA	83	3.9
Kuwait	8	3	11	2	2	4	2.8
Qatar	1	4	5	0	2	2	2.5
Yemen	164	80	244	72	37	109	2.2
Oman	69	19	88	50	5	55	1.6
Sudan	1189	60	1249	817	65	882	1.4
Morocco	158	284	442	161	187	348	1.3
Tunisia	27	7	34	18	13	31	1.1
Djibouti	78	NA	78	125	NA	125	0.6
Syria	21	19	40	77	9	86	0.5
Palestine	0	0	0	0	0	0	

a Gender ratios (male/female) calculated from data provided in table 2, Bozicevic *et al*. [2].

There are some exceptions in the region to the above predominance of men among HIV infections. In Djibouti, for example, women have already overtaken men in contribution to HIV cases, and there are increasing proportions of females among HIV/AIDS cases in Algeria, Sudan and Yemen [5]. In Morocco, the epidemic is also increasingly becoming feminized, and a study using the mode of transmission model revealed an important finding relevant to the rest of the region – that while 90% of the HIV risk behaviour in Morocco is practiced by men, HIV incidence among women is almost equal to that of men [7]. In that study, 70.7% of infections among women were due to an infected spouse.

Given prevailing health systems and policies in the MENA region, there are many reasons that HIV among women may be undetected and underreported. Hermez and colleagues point out that the region is characterized by a predominance of mandatory rather than voluntary testing for HIV, that provider-initiated testing is rare in the region and that as of 2010, only five countries mandated testing of pregnant women [8]. UNAIDS reports that access to treatment for pregnant HIV-positive women is low in the region [9]. This is beginning to change, however. Morocco, for example, in 2012 initiated a four-year plan to eliminate mother-to-child transmission of HIV [10]. There may also be cultural sensitivities to voluntary provider-initiated testing by women, as suggested by a study in Lebanon that Obstetrician–Gynaecologists rarely address sexual health with their patients [11]. Although there is little gender-disaggregated data on users of Voluntary Counselling and Testing (VCT) services in the region, a study in Lebanon found that women represented only 30% of VCT users in 2008 [12]. A recent qualitative study among HIV-positive women in Egypt found that these women lacked information about HIV through reproductive health services, and had never been tested before their husbands were tested positive for HIV [13]. An earlier study in Egypt found that two-thirds of HIV-positive men sampled did not inform their wives on their diagnosis [14]. Moreover, most countries in the region lack systems of regular STI case reporting, making it challenging to assess levels of STIs and HIV among women.

**Figure 1:**
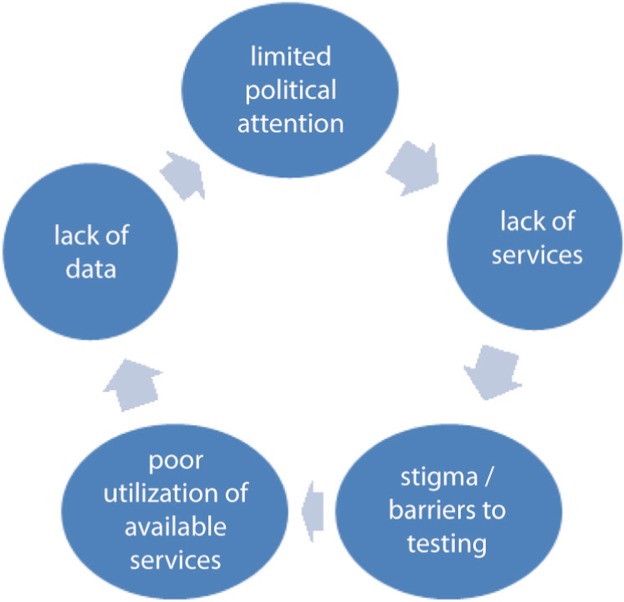
The vicious cycle increasing women's susceptibility to HIV in the MENA region.

Given these constraints, it is difficult to have an accurate estimate about HIV infection among women, and HIV may be under-detected, depriving HIV-positive women of access to treatment that is provided free in the region. The important structural determinants of HIV in the region including the disruptive effects of conflict [10] and women's economic dependence on men (in a region with the lowest female labour force participation rates in the world) may further compound these problems. Thus, the region may be in a vicious cycle whereby lack of policy attention to the issue leads to lack of services, compounded by stigma and social barriers to testing, leading in turn to a lack of data on HIV in women (see Figure 1).

There are some nascent initiatives reaching HIV-positive women in the region, including the formation of a regional network of HIV-positive women, MENA-ROSA [10,15] and efforts in Algeria to help HIV-positive women re-enter the workforce [10]. Nevertheless, there is an urgent need for context specific research (since the dynamics of the epidemic vary from country to country) and the specific needs of at-risk women in accessing testing and prevention, and, for those who test positive, treatment and sexual and reproductive health services. But even before research can be generated, sufficient evidence is available underscoring the urgent need for: gender-disaggregated data on HIV testing, case reporting and HIV-related statistics; increasing access to VCT services with specific attention to gender; encouraging voluntary provider-initiated testing among women; increasing efforts towards partner notification and access to treatment; and addressing the sexual and reproductive health needs of HIV-positive women. Given the epidemic patterns in the region, preventing HIV will require concerted efforts to address the populations most at risk, most of whom are likely to be men, but it is a public health imperative to address the needs and risks of their female partners.
